# Dibromido(6-methyl-2,2′-bipyridine-κ^2^ *N*,*N*′)zinc(II)

**DOI:** 10.1107/S1600536810035658

**Published:** 2010-09-11

**Authors:** Khadijeh Kalateh, Roya Ahmadi, Vahid Amani

**Affiliations:** a Islamic Azad University, Shahr-e-Rey Branch, Tehran, Iran

## Abstract

In the title compound, [ZnBr_2_(C_11_H_10_N_2_)], the Zn^II^ atom is four-coordinated in a distorted tetra­hedral configuration by two N atoms from a 6-methyl-2,2′-bipyridine ligand and two terminal Br atoms. Weak inter­molecular C—H⋯Br hydrogen bonds and π–π stacking inter­actions between the pyridine rings [centroid–centroid distances = 3.763 (5) and 3.835 (6) Å] contribute to crystal-packing effects.

## Related literature

For unusual coordination geometries on transition metal atoms, see: Beeston *et al.* (1998 ▶), Meyer *et al.* (1999 ▶); For related literature, see: Ahmadi *et al.* (2009 ▶); Ahmadi, Ebadi *et al.* (2008 ▶); Ahmadi, Kalateh *et al.* (2008 ▶); Alizadeh *et al.* (2009 ▶); Amani *et al.* (2009 ▶); Newkome *et al.* (1982 ▶); Onggo *et al.* (1990 ▶, 2005 ▶).
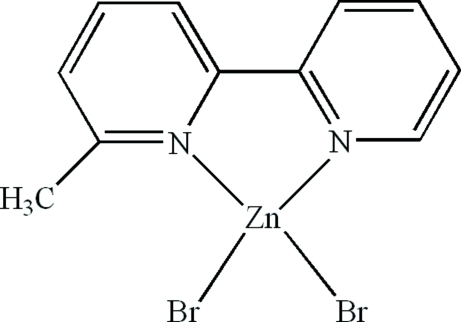


## Experimental

### 

#### Crystal data

[ZnBr_2_(C_11_H_10_N_2_)]
*M* *_r_* = 395.40
Monoclinic, 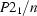

*a* = 7.6445 (7) Å
*b* = 9.7487 (11) Å
*c* = 17.8347 (18) Å
β = 96.972 (8)°
*V* = 1319.3 (2) Å^3^
*Z* = 4
Mo *K*α radiation
μ = 7.89 mm^−1^
*T* = 298 K
0.46 × 0.30 × 0.15 mm

#### Data collection

Bruker SMART CCD area-detector diffractometer
Absorption correction: multi-scan (*SADABS*; Sheldrick, 2003 ▶) *T*_min_ = 0.076, *T*_max_ = 0.310
15389 measured reflections
3567 independent reflections
2498 reflections with *I* > 2σ(*I*)
*R*_int_ = 0.119

#### Refinement

*R*[*F* ^2^ > 2σ(*F* ^2^)] = 0.086
*wR*(*F* ^2^) = 0.207
*S* = 1.13
3567 reflections
145 parameters
H-atom parameters constrained
Δρ_max_ = 2.14 e Å^−3^
Δρ_min_ = −1.14 e Å^−3^

### 

Data collection: *SMART* (Bruker, 1998 ▶); cell refinement: *SAINT* (Bruker, 1998 ▶); data reduction: *SAINT*; program(s) used to solve structure: *SHELXTL* (Sheldrick, 2008 ▶); program(s) used to refine structure: *SHELXTL*; molecular graphics: *ORTEP-3* (Farrugia, 1997 ▶); software used to prepare material for publication: *WinGX* (Farrugia, 1999 ▶).

## Supplementary Material

Crystal structure: contains datablocks global, I. DOI: 10.1107/S1600536810035658/jj2041sup1.cif

Structure factors: contains datablocks I. DOI: 10.1107/S1600536810035658/jj2041Isup2.hkl

Additional supplementary materials:  crystallographic information; 3D view; checkCIF report

## Figures and Tables

**Table 1:** Hydrogen-bond geometry (Å, °)

*D*—H⋯*A*	*D*—H	H⋯*A*	*D*⋯*A*	*D*—H⋯*A*
C1—H1*C*⋯Br1^i^	0.96	2.86	3.805 (14)	169
C8—H8⋯Br1^ii^	0.93	2.93	3.812 (12)	159

Symmetry codes: (i) 
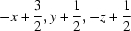
; (ii) 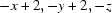.

**Fig. 1.**
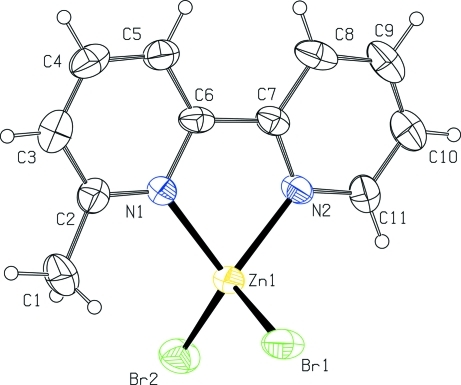
The molecular structure of the title molecule, [Zn(C11H10N2)Br2], with the atom-numbering scheme. Displacement ellipsoids are drawn at the 30% probability level.

**Fig. 2.**
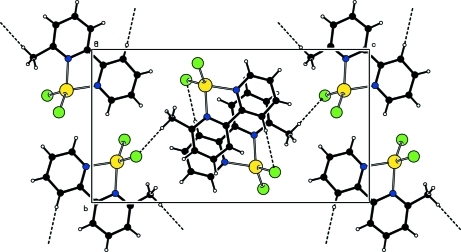
Unit-cell packing diagram for [Zn(C11H10N2)Br2]. Dashed lines indicate weak C—H···Br intermolecular interactions.

## supplementary crystallographic information

### Comment

Sterically hindered ligands such as 6-Methyl-2, 2'-bipyridine (6-mbipy) often convey unusual coordination geometries or oxidation states on transition metal centers (Beeston *et al.*, 1998; Meyer *et al.* 1999). Numerous complexes with 6-mbipy have been prepared, such as that of mercury (Ahmadi, Ebadi *et al.*, 2008), platin (Amani *et al.*, 2009), lead (Ahmadi *et al.*, 2009), palladium (Newkome *et al.*, 1982), ruthenium (Onggo, Scudder *et al.*, 2005) and iron (Onggo, Hook *et al.*, 1990). Here, we report the synthesis and structure of the title compound, [Zn(C_11_H_10_N_2_)Br_2_].

In the title compound (Fig. 1), the Zn^II^ atom is four-coordinated in a distorted tetrahedral configuration by two N atoms from one 6-methyl-2,2'-bipyridine and two terminal Br atoms. The Zn—N and Zn—Br bond lengths and angles are within the normal range of [ZnCl_2_(6-mbpy)], (Ahmadi, Kalateh *et al.*, 2008) and [ZnBr_2_(6,6'-dmbpy)], (Alizadeh *et al.*, 2009) [where 6,6'-dmbpy is 6,6'-dimethyl-2, 2'-bipyridine] respectively.

In the crystal structure, weak intermolecular C—H···Br hydrogen bonds (Table 2) and π···π stacking interactions (Fig. 2, Table 1) between the pyridine rings,*Cg*1—*Cg*2 and *Cg*2—*Cg*3 contribute to crystal packing effects [where *Cg*1, *Cg*2 and *Cg*3 are centroids of the rings (Zn1/N1/C6—C7/N2), (N1/C2—C6) and (N2/C7—C11), respectively].

### Experimental

For the preparation of the title compound, a solution of 6-methyl-2,2'-bipyridine (0.16 g, 0.15 ml 0.94 mmol) in methanol (10 ml) was added to a solution of ZnBr_2_ (0.21 g, 0.94 mmol) in acetonitrile (30 ml) and the resulting colorless solution was stirred for 20 min at 313 K. This solution was left to evaporate slowly at room temperature. After one week, colorless prismatic crystals of the title compound were isolated (yield 0.28 g, 75.3%).

### Refinement

All H atoms were positioned geometrically, with C—H = 0.93Å for aromatics H, C—H = 0.96Å for methyl and constrained to ride on their parent atoms, with *U*_iso_(H) = 1.2*U*_eq_.  High values for Δρ are related to the poor quality of the crystals.

### Crystal data

**Table:**

[ZnBr_2_(C_11_H_10_N_2_)]	*F*(000) = 760
*M**_r_* = 395.40	*D*_x_ = 1.991 Mg m^−^^3^
Monoclinic, *P*2_1_/*n*	Mo *K*α radiation, λ = 0.71073 Å
Hall symbol: -P 2yn	Cell parameters from 985 reflections
*a* = 7.6445 (7) Å	θ = 2.3–29.4°
*b* = 9.7487 (11) Å	µ = 7.89 mm^−^^1^
*c* = 17.8347 (18) Å	*T* = 298 K
β = 96.972 (8)°	Prism, colorless
*V* = 1319.3 (2) Å^3^	0.46 × 0.30 × 0.15 mm
*Z* = 4	

### Data collection

**Table:**

Bruker SMART CCD area-detector diffractometer	3567 independent reflections
Radiation source: fine-focus sealed tube	2498 reflections with *I* > 2σ(*I*)
graphite	*R*_int_ = 0.119
φ and ω scans	θ_max_ = 29.4°, θ_min_ = 2.3°
Absorption correction: multi-scan (*SADABS*; Sheldrick, 2003)	*h* = −8→10
*T*_min_ = 0.076, *T*_max_ = 0.310	*k* = −13→13
15389 measured reflections	*l* = −24→24

### Refinement

**Table:**

Refinement on *F*^2^	Primary atom site location: structure-invariant direct methods
Least-squares matrix: full	Secondary atom site location: difference Fourier map
*R*[*F*^2^ > 2σ(*F*^2^)] = 0.086	Hydrogen site location: inferred from neighbouring sites
*wR*(*F*^2^) = 0.207	H-atom parameters constrained
*S* = 1.13	*w* = 1/[σ^2^(*F*_o_^2^) + (0.0565*P*)^2^ + 8.4252*P*] where *P* = (*F*_o_^2^ + 2*F*_c_^2^)/3
3567 reflections	(Δ/σ)_max_ = 0.002
145 parameters	Δρ_max_ = 2.14 e Å^−^^3^
0 restraints	Δρ_min_ = −1.14 e Å^−^^3^

### Special details

**Table:**

Geometry. All e.s.d.'s (except the e.s.d. in the dihedral angle between two l.s. planes) are estimated using the full covariance matrix. The cell e.s.d.'s are taken into account individually in the estimation of e.s.d.'s in distances, angles and torsion angles; correlations between e.s.d.'s in cell parameters are only used when they are defined by crystal symmetry. An approximate (isotropic) treatment of cell e.s.d.'s is used for estimating e.s.d.'s involving l.s. planes.
Refinement. Refinement of *F*^2^ against ALL reflections. The weighted *R*-factor *wR* and goodness of fit *S* are based on *F*^2^, conventional *R*-factors *R* are based on *F*, with *F* set to zero for negative *F*^2^. The threshold expression of *F*^2^ > σ(*F*^2^) is used only for calculating *R*-factors(gt) *etc*. and is not relevant to the choice of reflections for refinement. *R*-factors based on *F*^2^ are statistically about twice as large as those based on *F*, and *R*- factors based on ALL data will be even larger.

### Fractional atomic coordinates and isotropic or equivalent isotropic displacement parameters (Å^2^)

**Table:**

	*x*	*y*	*z*	*U*_iso_*/*U*_eq_	
C1	0.651 (2)	0.9482 (15)	0.2106 (6)	0.099 (5)	
H1A	0.7590	0.9042	0.2302	0.119*	
H1B	0.5594	0.8805	0.2012	0.119*	
H1C	0.6183	1.0135	0.2468	0.119*	
C2	0.6740 (14)	1.0200 (10)	0.1390 (6)	0.063 (2)	
C3	0.6519 (15)	1.1604 (12)	0.1292 (7)	0.074 (3)	
H3	0.6227	1.2153	0.1685	0.089*	
C4	0.6738 (15)	1.2162 (11)	0.0608 (8)	0.075 (3)	
H4	0.6559	1.3097	0.0527	0.090*	
C5	0.7221 (13)	1.1355 (10)	0.0041 (6)	0.063 (2)	
H5	0.7415	1.1739	−0.0419	0.076*	
C6	0.7415 (11)	0.9971 (9)	0.0160 (5)	0.051 (2)	
C7	0.7873 (11)	0.8977 (10)	−0.0412 (5)	0.0509 (19)	
C8	0.8184 (14)	0.9373 (12)	−0.1129 (6)	0.070 (3)	
H8	0.8168	1.0293	−0.1268	0.084*	
C9	0.8523 (16)	0.8339 (15)	−0.1639 (6)	0.080 (3)	
H9	0.8735	0.8568	−0.2126	0.096*	
C10	0.8542 (18)	0.7025 (14)	−0.1426 (6)	0.080 (3)	
H10	0.8736	0.6339	−0.1768	0.097*	
C11	0.8280 (15)	0.6692 (12)	−0.0710 (6)	0.072 (3)	
H11	0.8344	0.5776	−0.0563	0.087*	
N1	0.7182 (10)	0.9406 (7)	0.0849 (4)	0.0508 (17)	
N2	0.7930 (10)	0.7647 (8)	−0.0208 (4)	0.0530 (17)	
Br1	1.04853 (15)	0.69883 (13)	0.15998 (7)	0.0756 (4)	
Br2	0.54078 (15)	0.58954 (13)	0.11809 (7)	0.0767 (4)	
Zn1	0.76827 (14)	0.73342 (11)	0.09099 (6)	0.0525 (3)	

### Atomic displacement parameters (Å^2^)

**Table:**

	*U*^11^	*U*^22^	*U*^33^	*U*^12^	*U*^13^	*U*^23^
C1	0.147 (13)	0.102 (10)	0.056 (6)	0.016 (9)	0.040 (8)	−0.011 (6)
C2	0.063 (6)	0.057 (5)	0.068 (6)	0.004 (5)	0.010 (5)	−0.004 (5)
C3	0.061 (6)	0.070 (7)	0.088 (8)	0.006 (5)	0.001 (5)	−0.018 (6)
C4	0.071 (7)	0.051 (5)	0.102 (9)	0.003 (5)	0.005 (6)	0.013 (6)
C5	0.063 (6)	0.056 (5)	0.070 (6)	0.003 (5)	0.008 (5)	0.015 (5)
C6	0.038 (4)	0.058 (5)	0.056 (5)	−0.005 (4)	−0.001 (3)	0.021 (4)
C7	0.045 (4)	0.063 (5)	0.043 (4)	−0.006 (4)	0.002 (3)	0.009 (4)
C8	0.060 (6)	0.089 (7)	0.058 (6)	−0.018 (5)	0.002 (5)	0.028 (5)
C9	0.076 (7)	0.122 (11)	0.046 (5)	−0.016 (7)	0.018 (5)	−0.005 (6)
C10	0.091 (8)	0.098 (9)	0.056 (6)	−0.006 (7)	0.021 (6)	−0.010 (6)
C11	0.081 (7)	0.073 (7)	0.068 (6)	0.007 (6)	0.029 (6)	−0.004 (5)
N1	0.052 (4)	0.050 (4)	0.052 (4)	0.004 (3)	0.015 (3)	0.008 (3)
N2	0.059 (4)	0.059 (4)	0.042 (3)	−0.001 (4)	0.012 (3)	0.008 (3)
Br1	0.0576 (6)	0.0870 (8)	0.0815 (7)	0.0001 (5)	0.0052 (5)	0.0382 (6)
Br2	0.0673 (7)	0.0768 (7)	0.0878 (8)	−0.0131 (5)	0.0173 (6)	0.0197 (6)
Zn1	0.0560 (6)	0.0509 (6)	0.0528 (6)	0.0038 (5)	0.0149 (4)	0.0129 (4)

### Geometric parameters (Å, °)

**Table:**

C1—C2	1.486 (16)	C7—N2	1.346 (11)
C1—H1A	0.9600	C7—C8	1.383 (12)
C1—H1B	0.9600	C8—C9	1.402 (17)
C1—H1C	0.9600	C8—H8	0.9300
C2—N1	1.312 (12)	C9—C10	1.335 (18)
C2—C3	1.388 (15)	C9—H9	0.9300
C3—C4	1.364 (17)	C10—C11	1.355 (15)
C3—H3	0.9300	C10—H10	0.9300
C4—C5	1.368 (16)	C11—N2	1.342 (13)
C4—H4	0.9300	C11—H11	0.9300
C5—C6	1.370 (13)	N1—Zn1	2.057 (7)
C5—H5	0.9300	N2—Zn1	2.048 (7)
C6—N1	1.378 (10)	Br1—Zn1	2.3617 (16)
C6—C7	1.480 (13)	Br2—Zn1	2.3300 (15)
			
Cg1···Cg2^i^	3.762 (5)	Cg2···Cg3^ii^	3.835 (6)
			
C2—C1—H1A	109.5	C7—C8—C9	117.7 (10)
C2—C1—H1B	109.5	C7—C8—H8	121.2
H1A—C1—H1B	109.5	C9—C8—H8	121.2
C2—C1—H1C	109.5	C10—C9—C8	120.1 (10)
H1A—C1—H1C	109.5	C10—C9—H9	120.0
H1B—C1—H1C	109.5	C8—C9—H9	120.0
N1—C2—C3	121.8 (10)	C9—C10—C11	120.0 (11)
N1—C2—C1	115.0 (9)	C9—C10—H10	120.0
C3—C2—C1	123.2 (10)	C11—C10—H10	120.0
C4—C3—C2	118.7 (11)	N2—C11—C10	121.8 (11)
C4—C3—H3	120.7	N2—C11—H11	119.1
C2—C3—H3	120.7	C10—C11—H11	119.1
C3—C4—C5	120.3 (10)	C2—N1—C6	119.5 (8)
C3—C4—H4	119.8	C2—N1—Zn1	127.1 (6)
C5—C4—H4	119.8	C6—N1—Zn1	113.3 (6)
C4—C5—C6	119.1 (10)	C11—N2—C7	119.4 (8)
C4—C5—H5	120.5	C11—N2—Zn1	126.6 (7)
C6—C5—H5	120.5	C7—N2—Zn1	113.8 (6)
C5—C6—N1	120.5 (9)	N2—Zn1—N1	80.9 (3)
C5—C6—C7	124.6 (8)	N2—Zn1—Br2	116.8 (2)
N1—C6—C7	114.9 (8)	N1—Zn1—Br2	117.6 (2)
N2—C7—C8	121.0 (9)	N2—Zn1—Br1	110.1 (2)
N2—C7—C6	116.5 (7)	N1—Zn1—Br1	108.6 (2)
C8—C7—C6	122.4 (9)	Br2—Zn1—Br1	117.30 (6)
			
N1—C2—C3—C4	1.2 (17)	C5—C6—N1—Zn1	−176.7 (7)
C1—C2—C3—C4	−179.0 (12)	C7—C6—N1—Zn1	3.4 (9)
C2—C3—C4—C5	−2.1 (18)	C10—C11—N2—C7	−1.4 (17)
C3—C4—C5—C6	2.5 (17)	C10—C11—N2—Zn1	−175.1 (9)
C4—C5—C6—N1	−2.0 (15)	C8—C7—N2—C11	−0.5 (14)
C4—C5—C6—C7	177.9 (9)	C6—C7—N2—C11	177.8 (9)
C5—C6—C7—N2	−177.1 (9)	C8—C7—N2—Zn1	174.0 (7)
N1—C6—C7—N2	2.9 (11)	C6—C7—N2—Zn1	−7.7 (10)
C5—C6—C7—C8	1.2 (14)	C11—N2—Zn1—N1	−178.6 (9)
N1—C6—C7—C8	−178.8 (8)	C7—N2—Zn1—N1	7.4 (6)
N2—C7—C8—C9	1.3 (15)	C11—N2—Zn1—Br2	−62.2 (9)
C6—C7—C8—C9	−176.9 (9)	C7—N2—Zn1—Br2	123.7 (6)
C7—C8—C9—C10	−0.1 (17)	C11—N2—Zn1—Br1	74.8 (9)
C8—C9—C10—C11	−2(2)	C7—N2—Zn1—Br1	−99.2 (6)
C9—C10—C11—N2	3(2)	C2—N1—Zn1—N2	176.6 (9)
C3—C2—N1—C6	−0.8 (15)	C6—N1—Zn1—N2	−5.8 (6)
C1—C2—N1—C6	179.5 (10)	C2—N1—Zn1—Br2	61.0 (9)
C3—C2—N1—Zn1	176.7 (8)	C6—N1—Zn1—Br2	−121.3 (5)
C1—C2—N1—Zn1	−3.0 (14)	C2—N1—Zn1—Br1	−75.2 (8)
C5—C6—N1—C2	1.2 (13)	C6—N1—Zn1—Br1	102.5 (6)
C7—C6—N1—C2	−178.7 (8)		

Symmetry codes: (i) −*x*+1, −*y*+2, −*z*; (ii) −*x*+2, −*y*+2, −*z*.

### Hydrogen-bond geometry (Å, °)

**Table:**

*D*—H···*A*	*D*—H	H···*A*	*D*···*A*	*D*—H···*A*
C1—H1C···Br1^iii^	0.96	2.86	3.805 (14)	169
C8—H8···Br1^ii^	0.93	2.93	3.812 (12)	159

Symmetry codes: (iii) −*x*+3/2, *y*+1/2, −*z*+1/2; (ii) −*x*+2, −*y*+2, −*z*.
